# Challenges in Communicating a Genetic Diagnosis

**DOI:** 10.3390/children10040672

**Published:** 2023-03-31

**Authors:** Francisco Cammarata-Scalisi, Colin Eric Willoughby, Vito Romano, Michele Callea

**Affiliations:** 1 Servicio de Pediatría, Hospital Regional de Antofagasta, Azapa 5935, Chile; francocammarata19@gmail.com; 2 Genomic Medicine, Biomedical Sciences Research Institute, Ulster University, Coleraine BT52 1SA, UK; 3 Eye Unit, Department of Medical and Surgical Specialties, Radiological Sciences and Public Health, University of Brescia, Viale Europa 15, 25123 Brescia, Italy; 4 Pediatric Dentistry and Special Dental Care Unit, Meyer Children’s Hospital IRCCS, 50139 Florence, Italy

Communicating the diagnosis of a genetic entity/rare disease to a patient or their parents is a complex process; it requires the doctor, pediatrician, or geneticist to display good communication skills and knowledge in a moment of uncertainty and disorientation for the family group, and sometimes in an inappropriate environment or under time constraints.

A genetic diagnosis can be made for patients at different life stages: prenatal, at birth, and later, and even in adulthood, as is the case with some types of cancer or neurodegenerative entities. If diagnosis occurs before birth, it must be confirmed at the time of birth. This requires that staff be responsible in preparing to communicate the information, which should ideally be transmitted to both parents at the same time. Furthermore, if it is a presumptive clinical diagnosis, or if it has been confirmed through a genetic study, this must be explained in a way that is easy to understand.

Today, it is common for family members to look for information on the internet, so it is necessary to emphasize that this information must be obtained from a reliable source, such as expert publications; moreover, it must be emphasized that the clinical case being described represents an individual, and may not be relevant to other individuals with the same genetic entity. Additionally, it must be highlighted that this process should preferably take place in person and not via phone call or message. The professional must address doubts in a precise manner based on up-to-date information and provide a description of the genetic entity; additionally, they may provide information about support groups or on local or national foundations.

Throughout this process, it is necessary to create a relationship based on trust and empathy. The professional should pause if a family member begins to cry, which forms part of the acceptance process and subsequent learning, and they must demonstrate understanding of the individual’s feelings. It is necessary to avoid inappropriate terms that do not contribute to the communication process. Likewise, it is necessary for the professional to determine whether the parents or family know about the genetic condition, which will provide an opportunity to correct any misconceptions or alleviate doubts. The answers given by the professional must be communicated clearly and calmly and end with a brief summary of the clinical characteristics of the patient’s condition; in doing so, they must maintain an appropriate balance of information on aspects of the diagnosis that must be considered without providing too much information, as this would be exhausting for both parties. These steps represent a mutual learning process for all parties involved, in which time is an important factor.

Considering the different aspects of the genetic entity under study, its symptoms and clinical signs, and complications that may occur, specialist control is required, and therapeutic strategies for interdisciplinary treatment must be developed. All this information must be communicated, which requires commitment from the parents or the patient to prepare themselves for the challenges raised by the genetic diagnosis. The patient’s existing abilities and skills are important positives in instances where psychomotor and intellectual development may be compromised.

All communication of genetic diagnoses must be accompanied by timely genetic counseling, either for parents who wish to have more children or for patients who wish to know the risks to their offspring. In clinical genetics, consultations not only involve investigating a genetic diagnosis and genetic counseling, but must also involve monitoring of the patient’s phenotypic evolution, which constitutes an important pillar in the patient’s interdisciplinary management.

In this Special Issue, the authors explain the complexity of diagnosing and communicating diagnoses of rare genetic entities, such as Noonan Syndrome, MECP2 duplication syndromes, Nieman-Pick type C disease, Pompe disease, hypohidrotic ectodermal dysplasia, retropharyngeal synovial cell carcinoma, 22q.11.2 deletion syndrome, and Pfeiffer syndrome [1,2,3,4,5,6,7,8].

## References

[1] Janas-Naze A., Malkiewicz K., Zhang W. (2022). Clinical Findings in Children with Noonan Syndrome—A 17-Year Retrospective Study in an Oral Surgery Center. Children.

[2] Ta D., Downs J., Baynam G., Wilson A., Richmond P., Leonard H. (2022). Medical Comorbidities in *MECP2* Duplication Syndrome: Results from the International *MECP2* Duplication Database. Children.

[3] Kraus D., Abdelrahim H., Waisbourd-Zinman O., Domin E., Zeharia A., Staretz-Chacham O. (2022). Elevated Alpha-Fetoprotein in Infantile-Onset Niemann-Pick Type C Disease with Liver Involvement. Children.

[4] Marques J.S. (2022). The Clinical Management of Pompe Disease: A Pediatric Perspective. Children.

[5] Callea M., Bignotti S., Semeraro F., Cammarata-Scalisi F., El-Feghaly J., Morabito A., Romano V., Willoughby C.E. (2022). Extended Overview of Ocular Phenotype with Recent Advances in Hypohidrotic Ectodermal Dysplasia. Children.

[6] Jassem-Bobowicz J.M., Sokołowska E.M., Hinca K.M., Drążkowska I., Stefańska K. (2022). From the Difficult Airway Management to Diagnosis of Retropharyngeal Synovial Cell Carcinoma. Children.

[7] Cortés-Martín J., Peñuela N.L., Sánchez-García J.C., Montiel-Troya M., Díaz-Rodríguez L., Rodríguez-Blanque R. (2022). Deletion Syndrome 22q11.2: A Systematic Review. Children.

[8] Jang M.J., Ahn M.B. (2022). Effect of Growth Hormone Therapy on a 4-Year-Old Girl with Pfeiffer Syndrome and Short Stature: A Case Report. Children.

